# Socioeconomic Inequalities in Secondhand Smoke Exposure at Home and at Work in 15 Low- and Middle-Income Countries

**DOI:** 10.1093/ntr/ntv261

**Published:** 2015-11-25

**Authors:** Gaurang P. Nazar, John Tayu Lee, Monika Arora, Christopher Millett

**Affiliations:** 1 ^1^ Health Promotion Division, Public Health Foundation of India, New Delhi, India;; 2 ^2^ Department of Non-Communicable Disease Epidemiology, Faculty of Epidemiology and Population Health, London School of Hygiene and Tropical Medicine, London, United Kingdom;; 3 ^3^ School of Public Health, Faculty of Medicine, Imperial College London, London, United Kingdom;; 4 ^4^ Saw Swee Hock School of Public Health, National University of Singapore, Singapore

## Abstract

**Introduction:**

In high-income countries, secondhand smoke (SHS) exposure is higher among disadvantaged groups. We examine socioeconomic inequalities in SHS exposure at home and at workplace in 15 low- and middle-income countries (LMICs).

**Methods:**

Secondary analyses of cross-sectional data from 15 LMICs participating in Global Adult Tobacco Survey (participants ≥ 15 years; 2008–2011) were used. Country-specific analyses using regression-based methods were used to estimate the magnitude of socioeconomic inequalities in SHS exposure: (1) Relative Index of Inequality and (2) Slope Index of Inequality.

**Results:**

SHS exposure at home ranged from 17.4% in Mexico to 73.1% in Vietnam; exposure at workplace ranged from 16.9% in Uruguay to 65.8% in Bangladesh. In India, Bangladesh, Thailand, Malaysia, Philippines, Vietnam, Uruguay, Poland, Turkey, Ukraine, and Egypt, SHS exposure at home reduced with increasing wealth (Relative Index of Inequality range: 1.13 [95% confidence interval [CI] 1.04–1.22] in Turkey to 3.31 [95% CI 2.91–3.77] in Thailand; Slope Index of Inequality range: 0.06 [95% CI 0.02–0.11] in Turkey to 0.43 [95% CI 0.38–0.48] in Philippines). In these 11 countries, and in China, SHS exposure at home reduced with increasing education. In India, Bangladesh, Thailand, and Philippines, SHS exposure at workplace reduced with increasing wealth. In India, Bangladesh, Thailand, Philippines, Vietnam, Poland, Russian Federation, Turkey, Ukraine, and Egypt, SHS exposure at workplace reduced with increasing education.

**Conclusion:**

SHS exposure at homes is higher among the socioeconomically disadvantaged in the majority of LMICs studied; at workplaces, exposure is higher among the less educated. Pro-equity tobacco control interventions alongside targeted efforts in these groups are recommended to reduce inequalities in SHS exposure.

**Implications:**

SHS exposure is higher among the socioeconomically disadvantaged groups in high-income countries. Comprehensive smoke-free policies are pro-equity for certain health outcomes that are strongly influenced by SHS exposure. Using nationally representative Global Adult Tobacco Survey (2008–2011) data from 15 LMICs, we studied socioeconomic inequalities in SHS exposure at homes and at workplaces. The study showed that in most LMICs, SHS exposure at homes is higher among the poor and the less educated. At workplaces, SHS exposure is higher among the less educated groups. Accelerating implementation of pro-equity tobacco control interventions and strengthening of efforts targeted at the socioeconomically disadvantaged groups are needed to reduce inequalities in SHS exposure in LMICs.

## Introduction

Exposure to secondhand smoke (SHS) has led to more than 600 000 deaths globally in 2010,^1^ with women and children bearing the maximum brunt of it.^2^ Adverse health outcomes among adults include cardiovascular and respiratory diseases; among children, SHS exposure causes low birth weight, sudden death, and middle ear infections.^3^ Eighty percent of the world’s smokers live in the low- and middle-income countries (LMICs), and SHS exposure in homes, workplaces, and other public places in many LMIC settings remains high.^4^ The World Health Organization (WHO) Framework Convention on Tobacco Control (FCTC) recommends comprehensive smoke-free policies to protect people from SHS.^5^

Socioeconomic status (SES) influences tobacco use with higher consumption among the poor and those with less education.^6^ A recent study examined socioeconomic inequalities in smoking in LMICs and suggested a similar overall negative gradient in smoking (smoking being most prevalent among low SES groups) to that documented in high-income countries (HICs).^7^ In LMICs, as in HICs, strong tobacco control policies, including smoke-free regulations, are responsible for changing social norms by promoting smoking as an unacceptable behavior, thereby protecting the nonsmokers from SHS exposure.^8,9^ Evidence from HICs suggests that SHS exposure is higher among the socioeconomically disadvantaged populations and occurs predominantly at homes and indoor workplaces^3^; however, there is little nationally representative data available from LMICs on socioeconomic inequalities in SHS exposure in these settings. Understanding the degrees of socioeconomic inequalities in SHS exposure can help identify opportunities to reduce inequalities in health. We examine socioeconomic inequalities in SHS exposure at home and at the workplace in 15 LMICs using nationally representative data from the Global Adult Tobacco Survey (GATS).

## Methods

### Study Design, Setting, and Data

We conducted secondary analysis on the GATS data, which is available freely on the Global Tobacco Surveillance System (GTSS) website^10^ of the Centers for Disease Control and Prevention (CDC). GATS is a nationally representative cross-sectional household survey of noninstitutionalized adults aged 15 years and above.^11^ It is considered to be the global standard for monitoring adult tobacco use and key tobacco control indicators. GATS employs a standardized survey methodology with a few country-specific variations in the questionnaire, and is designed to collect household as well as individual-level data. Multi-stage cluster sampling design is employed in GATS to select a nationally representative study sample. Between 2008 and 2011, the first round of GATS was implemented in 17 LMICs across five WHO regions.^10^ Country-specific, anonymous GATS data for 16 of the 17 LMICs (except Indonesia) was available from the CDC-GTSSData website. Poland and the Russian Federation are now classified as HICs by the World Bank; however, when the first round of GATS was conducted in these countries in 2009, they belonged to the upper middle-income category. Hence, for the purpose of our study, we treated them as middle-income countries. Further, we excluded Brazil from our data analyses because a key variable of interest, that is, the “wealth index,” was not comparable with that of other countries. Therefore, we included data from 15 countries in our analyses.

### Study Participants

We used individual-level data from the first round of GATS (2008–2011) for each of the 15 LMICs. Separate analyses were conducted to examine outcomes for “SHS exposure at home” and “SHS exposure at workplace.” In each country, analyses for the former outcome included all the GATS participants 15 years of age or older, while for the latter outcome, all GATS participants 15 years of age or older, who reported working indoors (or both indoors and outdoors) but outside their home were included as participants. Observations with missing values in the dependent or independent variables were dropped to obtain a final sample for each country.

### Measures

#### Dependent Variables

##### SHS Exposure at Home

A participant was considered to be exposed to SHS at home if he/she responded “daily,” “weekly,” or “monthly” to the question: How often does anyone smoke inside your home? If the participant responded “less than monthly” or “never” to the question, he/she was considered not exposed to SHS at home.

##### SHS Exposure at Workplace

A participant was considered to be exposed to SHS at workplace if he/she responded “yes” to the question: “During the past 30 days, did anyone smoke in indoor areas where you work?” If the participant responded “no” to the question, he/she was considered not exposed to SHS at workplace.

Participants who answered “don’t know” or “refused” to answer on either of the questions for the dependent variables were dropped from the study (see Supplementary Tables 1 and 2 for further details).

#### Independent Variables

##### Wealth Quintile

Using a previously validated method, that is, the inverse possession weighting approach, we computed a summary score from a list of household assets that the participants possessed (eg, electricity, flush toilet, car, and television).^12,13^ This approach uses the inverse of the proportion of households with an asset as a weight for the indicator, that is, higher weights are given to least possessed assets.^14^ We then divided the summary score into wealth quintiles, the lowest quintile being the poorest and the highest quintile being the richest.

##### Education

Education was grouped into three categories in all countries—completed education up to: primary level (no formal education, less than primary school completed, and primary school completed); secondary level (less than secondary school completed, secondary school completed, and higher secondary school completed); and tertiary level (college/university/postgraduate degree completed).

Other covariates included in the analyses were age group, gender, residence, and occupation. We also included the “geographic region” variable in the model for each country, whenever the variable was available. The countries in which “geographic region” variable was included were India, Thailand, China, Poland, and Ukraine.

### Statistical Methods

We used three regression-based methods to measure different dimensions of socioeconomic inequalities in SHS exposure: (1) Relative Index of Inequality (RII); (2) Slope Index of Inequality (SII); and (3) Adjusted Odds Ratio (*AOR*). The RII and SII are regression-based measures of inequalities that take into account SHS exposure across the entire socioeconomic distribution in the study population, whereas the *AOR* only compares relative differences of SHS exposure between the most affluent and the most deprived groups.^15,16^

To calculate RII and SII, individuals were cumulatively ranked (ranging from 0 to 1) according to their education and wealth status such that “0” represented the highest wealth/education level and “1” represented the lowest wealth/education level (RIDIT scores).^17^ RII provides a prevalence rate ratio while SII gives a prevalence rate difference of SHS exposure—between those participants having the lowest wealth/education levels and those having the highest wealth/education levels.^17^ We used a “modified Poisson” approach, as suggested by Zou to compute SII and RII, which provides more robust estimates as compared to using the binary approach.^18^ All analyses for calculations of RII and SII were adjusted for age group and gender. Values of SII larger than 0 and RII values larger than 1 indicate that the poor are more likely to be exposed to SHS compared with the rich; similarly, the less educated are more likely to be exposed to SHS compared with the more educated.

Further, we included interaction terms ([RIDIT scores for wealth levels × gender] and [RIDIT scores for education levels × gender]) in the country-specific generalized linear models to assess if inequalities in SHS exposure at home and at the workplace differed significantly by gender. We found that in the majority of these 15 countries, gender was not an effect-modifier. However, considering the findings of previously published studies,^4^ and our descriptive findings which suggest that gender differences exist in SHS exposure, we conducted disaggregated analysis by gender.

We also ran country-specific multiple logistic regression models to estimate the relationship between SES and SHS exposure (at home and at the workplace) and calculated *AOR*s comparing the lowest (the reference group) and the higher wealth quintiles, adjusted for age group, gender, residence, education, occupation, and region (for countries in which the variable was available). We tested for multicollinearity between all the covariates adjusted for in the analysis for each country. The multicollinearity diagnostics (variance inflation factor, VIF) were all less than 5, indicating that the assumption of reasonable independence among predictor variables was met. As SII and RII are more robust measures of inequality compared with the *AOR*, we present only those estimates in this paper. Moreover, the country-specific *AOR* estimates (Supplementary Figures 1 and 2) were broadly consistent with the SII and RII estimates in the majority of the countries studied.

Sampling weights (STATA svy: command) were used to account for the complex, multi-stage design of the GATS survey throughout the analyses. All the statistical analyses were performed using STATA version 13.1 (StataCorp LP, Texas). Exemption from ethics review for using anonymous secondary data freely available in public domain was obtained from the Research Ethics Committee at the London School of Hygiene and Tropical Medicine and Institutional Ethics Committee at Public Health Foundation of India.

## Results

### Descriptive Statistics

Table 1 shows that the number of participants ranged from 4091 in Malaysia to 67 006 in India. The percentage of missing values was generally low (less than 5%) for all the 15 countries studied and ranged from 0.1% in Uruguay to 3.7% in Malaysia. SHS exposure at home ranged from 17.4% in Mexico to 73.1% in Vietnam. In India, Bangladesh, Thailand, Malaysia, the Philippines, and Vietnam, the proportion of participants exposed to SHS at home was higher among poorer participants compared with that of richer participants. In India, Bangladesh, Thailand, Malaysia, the Philippines, Vietnam, Uruguay, and Turkey, the proportion of participants exposed to SHS at home was higher among those with lower education compared to those with higher education.

**Table 1.** SHS Exposure at Home Among GATS Participants (2008–2011)

Weighted %															
	SEAR			WPR				AMR		EUR					EMR
	India *N* = 67 006	Bangladesh *N* = 9323	Thailand *N* = 20 437	China^a^ *N* = 13 302	Malaysia *N* = 4091	Philippines *N* = 9578	Vietnam *N* = 9866	Mexico *N* = 13 530	Uruguay *N* = 5576	Poland^b^ *N* = 7640	Romania *N* = 4472	Russian Federation *N* = 11 321	Turkey^c^ *N* = 8900	Ukraine *N* = 8092	Egypt *N* = 20 443
Age group (y)															
≥15 to ≤29	39.9	53.3	36.6	68.8	42.2	53.9	75.6	18.5	44.8	46.1	46.1	36.9	64.1	27.4	64.8
≥30 to ≤44	39.8	57.8	32.2	67.3	38.0	54.9	74.6	16.8	32.5	43.4	36.5	37.6	57.2	26.0	62.2
≥45 to ≤59	40.3	54.6	31.2	69.1	36.0	55.6	71.9	17.4	33.5	50.1	38.8	35.8	53.1	25.4	61.9
≥60	39.9	51.4	32.0	61.8	30.8	52.4	64.0	14.9	22.4	36.1	20.9	26.5	39.3	14.5	53.7
Gender															
Male	40.6	58.2	37.4	70.5	43.2	58.1	77.2	17.3	36.8	45.0	37.8	36.7	56.2	25.3	61.3
Female	39.2	51.2	29.2	63.9	33.3	50.6	69.2	17.5	31.4	43.6	33.2	32.9	56.4	22.0	63.8
Residence															
Urban	29.4	44.6	25.4	60.1	35.7	43.4	63.3	19.0	34.0	42.9	40.9	35.8	55.1	24.2	57.5
Rural	44.3	58.2	36.7	73.4	45.4	65.2	77.4	11.6	33.7	46.6	28.5	31.1	59.2	21.9	66.8
Education															
Up to primary level	46.9	60.7	37.6	69.0	42.4	67.5	77.5	16.0	35.3	46.3	28.7	30.1	57.3	16.3	67.0
Up to secondary level	33.8	46.3	30.9	69.7	39.6	49.4	69.5	17.9	33.1	46.6	35.5	35.6	56.9	25.0	61.9
Up to tertiary level	20.4	21.3	15.4	51.3	25.7	31.1	45.6	20.0	31.0	31.3	39.1	33.5	47.6	20.3	47.5
Wealth quintile															
Q1 (poorest)	48.6	67.5	45.2	66.5	52.2	69.5	77.5	11.0	37.3	47.2	35.2	37.2	60.3	23.6	62.5
Q2	42.8	58.3	41.3	73.7	43.8	64.7	77.2	15.0	33.9	45.1	31.0	33.6	55.2	26.1	67.5
Q3	40.8	53.1	33.6	69.0	41.6	59.6	77.2	16.4	33.5	46.2	29.6	33.3	56.1	24.0	64.9
Q4	32.4	51.7	25.3	67.1	35.6	48.4	70.4	20.7	33.9	46.6	40.8	31.4	57.9	24.3	56.6
Q5 (most affluent)	22.9	38.9	13.5	60.6	28.0	34.6	58.9	19.0	32.8	37.2	36.7	37.3	53.1	20.0	53.9
Occupation															
Govt employee	27.6	34.3	19.0	69.7	29.2	44.0	56.1	16.6	33.7	46.0	36.8	33.1	55.4	21.8	53.0
Non-govt employee	40.5	37.7	36.0		43.2	53.7	65.4	19.7	37.2		40.5	40.6		29.7	64.3
Self-employed	44.1	63.4	34.7		47.0	62.1	78.7	16.3	37.2	44.5	34.8	33.6	60.0	28.0	67.5
Student	33.0	42.4	31.4	64.3	31.2	45.8	67.9	18.3	39.3	40.7	44.5	29.2	64.8	21.4	59.4
Others (retd & homemakers)	39.0	52.7	31.8	55.5	34.4	52.1	63.7	16.2	24.5	40.9	27.0	28.5	52.7	18.3	62.1
Unemployed	44.0	46.1	32.3	63.6	37.4	51.9	64.1	21.2	47.8	58.6	49.4	47.2	69.5	31.5	64.1
% reporting SHS exposure at home	39.9	54.7	33.2	67.3	38.5	54.3	73.1	17.4	33.9	44.2	35.4	34.6	56.3	23.5	62.6
% of missing cases	3.3	3.2	0.6	0.4	3.7	1.3	0.6	0.6	0.1	2.5	1.0	0.7	1.4	0.8	2.3

AMR = Region of the Americas; EMR = Eastern Mediterranean Region; EUR = European Region; GATS = Global Adult Tobacco Survey; SEAR = South-East Asia Region; SHS = secondhand smoke; WPR = Western Pacific Region.

^a^For China, it was not possible to distinguish between Government employee, nongovernment employee or self-employed as occupation categories have been defined differently as compared with other countries. Hence, the category “Employed” included all those participants who were either “Agriculture, Forestry, Fishery employee” or “Transportation, equipment operator” or, “Business or service industry employee” or “Leaders of organizations” or “Clerks” or “Specialized Technician” or “Medical and health personnel” or “Teaching staff” or “Soldier.”

^b^For Poland, it was not possible to distinguish between Government employee and nongovernment employee categories as occupation categories have been defined differently as compared with other countries. Hence only one category “employed” was considered to represent “employed in company/enterprise.”

^c^For Turkey, it was not possible to distinguish between Government employee and nongovernment employee categories as occupation categories have been defined differently as compared with other countries. Hence only one category “employed” was considered to represent “Paid employee.”

Table 2 shows that SHS exposure at workplace ranged from 16.9% in Uruguay to 65.8% in Bangladesh. For this outcome, the proportion of missing cases was generally low (less than 5%) for all the countries studied, except Bangladesh and Malaysia, and ranged from 0.2% in Uruguay to 11.4% in Malaysia. In 14 of the 15 countries studied (except China), the proportion of participants exposed to SHS at workplace was higher among those with lower education compared to those with higher education. Proportion of male participants exposed to SHS was notably higher as compared with females, particularly at workplaces.

**Table 2.** SHS Exposure at Workplace Among GATS Participants Employed Indoors and Outside Their Home (2008–2011)

Weighted %															
	SEAR			WPR				AMR		EUR					EMR
	India *N* =12 852	Bangladesh *N* = 1704	Thailand *N* = 5021	China^a^ *N* = 1859	Malaysia *N* = 996	Philippines *N* =2152	Vietnam *N* = 2419	Mexico *N* = 2082	Uruguay *N* = 1796	Poland^b^ *N* = 3030	Romania^c^ *N* = 1175	Russian Federation *N* = 5464	Turkey^b^ *N* = 2160	Ukraine *N* = 2761	Egypt *N* = 4490
Age group (y)															
≥15 to ≤29	31.4	64.6	20.8	63.7	40.9	26.6	49.3	18.4	18.3	31.0	38.8	38.4	37.8	32.3	59.5
≥30 to ≤44	29.5	65.5	27.1	62.9	38.9	33.1	61.3	17.8	16.7	33.5	32.9	37.2	36.9	34.1	60.7
≥45 to ≤59	29.9	68.7	29.9	72.5	41.6	37.5	58.2	22.2	16.8	35.4	34.1	35.2	38.4	30.3	59.7
≥60	30.6	67.0	38.2	64.5	34.0	45.1	69.4	19.1	12.7	39.2	24.6	25.9	34.8	33.9	56.8
Gender															
Male	32.7	70.3	33.2	77.1	46.6	38.5	68.9	22.2	21.9	41.6	36.8	48.2	40.5	43.3	61.6
Female	17.9	29.2	18.5	47.5	30.2	25.5	41.4	13.8	11.9	24.5	31.4	26.3	27.6	21.9	54.2
Residence															
Urban	27.9	59.7	23.4	65.2	42.2	24.9	52.8	18.9	16.8	31.4	35.2	37.2	35.6	32.5	59.1
Rural	32.7	69.9	28.3	65.9	32.5	45.9	59.1	18.0	20.3	38.3	31.9	32.9	45.1	32.5	60.9
Education															
Up to primary level	38.7	70.9	38.1	70.8	52.8	52.1	60.9	21.9	20.2	50.9	36.1	52.0	43.1	63.6	63.6
Up to secondary level	29.5	62.2	22.6	64.6	38.9	29.9	54.5	19.2	16.7	36.8		41.7	37.8	35.7	61.0
Up to tertiary level	20.4	54.4	18.9	65.5	38.8	21.2	39.5	15.9	10.8	23.1	30.0	31.5	25.4	26.2	55.2
Wealth quintile															
Q1 (poorest)	35.5	68.6	35.4	61.8	47.7	45.3	58.7	16.5	20.7	37.4	36.4	36.7	39.8	34.2	62.5
Q2	37.2	68.2	29.8	66.7	31.1	41.0	56.3	20.3	14.1	31.1	35.4	34.1	31.2	36.1	58.6
Q3	31.0	65.8	25.9	69.0	44.4	30.3	59.4	19.3	20.6	34.6	30.6	35.3	34.4	34.9	62.8
Q4	28.5	68.2	25.9	68.1	40.7	31.8	53.9	17.3	18.6	32.7	36.9	36.9	39.7	27.5	60.6
Q5 (most affluent)	23.6	60.3	17.3	60.3	38.7	26.4	53.5	19.4	13.9	33.7	32.8	38.7	41.8	33.1	54.9
Occupation															
Govt. employee	21.9	56.1	21.6	—	29.3	26.3	46.3	12.6	15.1	32.1	34.6	30.5	32.3	28.6	58.6
Non-govt employee	28.5	41.8	24.6	—	40.6	25.8	33.4	19.5	16.4		33.2	40.8		34.6	60.8
Self-employed	35.4	74.4	36.6	—	54.3	55.7	70.6	21.9	21.3	42.7	42.2	43.2	53.1	40.1	60.9
% exposed to SHS at workplace	30.3	65.8	26.0	65.5	40.1	32.2	56.2	18.8	16.9	33.6	34.3	36.3	37.4	32.5	59.9
% of missing cases	4.9	6.0	1.2	0.6	11.4	0.8	0.9	0.9	0.2	2.4	1.4	2.1	0.5	1.9	0.6

AMR = Region of the Americas; EMR = Eastern Mediterranean Region; EUR = European Region; GATS = Global Adult Tobacco Survey; LMICs = low- and middle-income countries; SEAR = South-East Asia Region; SHS = secondhand smoke; WPR = Western Pacific Region.

^a^Occupation categories in China differed from those of other LMICs. Five occupation categories were considered for China: Agriculture, forestry, fishery employee (78.5%); transportation equipment operator (61.7%); government, party, organization, company (73.3%); medical, health personnel (55.4%); teaching staff (54.8%). Not presented in table to maintain uniformity.

^b^For Poland and Turkey, categorization of occupation into “Government employee” and “Nongovernment employee” was not possible due to the way categories were defined hence the categories were merged into one category “Employed.”

^c^For Romania, the category educated up to primary level contained only one participant hence, this category was merged with educated up to secondary level for further analysis.

Supplementary Tables 1 and 2 show the numbers and proportion of missing cases in the dependent and independent variables for all the 15 countries for the outcomes “SHS exposure at home” and “SHS exposure at workplace,” respectively.

### Socioeconomic Inequalities in SHS Exposure at Home

Table 3 presents the RII and SII estimates and their 95% confidence intervals (CIs), respectively, for wealth and education inequality in SHS exposure at home. These comparisons are also shown graphically in Supplementary Figure 3. In 11 of the 15 countries studied (India, Bangladesh, Thailand, Malaysia, the Philippines, Vietnam, Uruguay, Poland, Turkey, Ukraine, and Egypt), the RII estimates were more than 1 and the SII estimates were more than 0, indicating that the poor are more likely to be exposed to SHS at home compared with the rich. There was substantial variation between the countries in SHS exposure at home by levels of wealth. The RII estimates ranged from 1.13 (95% CI 1.04–1.22) in Turkey to 3.31 (95% CI 2.91–3.77) in Thailand, while the SII estimates ranged from 0.06 (95% CI 0.02–0.11) in Turkey to 0.43 (95% CI 0.38–0.48) in the Philippines.

**Table 3.** Socioeconomic Inequality in SHS Exposure at Home

Region/Country	Wealth inequality						Education inequality					
	RII [95% CI]			SII [95% CI]			RII [95% CI]			SII [95% CI]		
	Males	Females	Total^a^	Males	Females	Total^b^	Males	Females	Total^a^	Males	Females	Total^b^
SEAR												
India (*N* = 67 006)	**1.99[1.78, 2.22]**	**2.28[2.04, 2.56]**	**2.12[1.94, 2.32]**	**0.28[0.24, 0.33]**	**0.32[0.28, 0.36]**	**0.30[0.27, 0.33]**	**2.41[2.18, 2.68]**	**3.09[2.70, 3.54]**	**2.65[2.43, 2.88]**	**0.36[0.32, 0.40]**	**0.42[0.37, 0.46]**	**0.38[0.35, 0.41]**
Bangladesh (*N* = 9323)	**1.92[1.66, 2.23]**	**1.67[1.45, 1.92]**	**1.81[1.62, 2.02]**	**0.38[0.30, 0.47]**	**0.27[0.20, 0.34]**	**0.32[0.27, 0.38]**	**2.3[1.93, 2.74]**	**1.98[1.61, 2.42]**	**2.14[1.87, 2.44]**	**0.47[0.38, 0.56]**	**0.35[0.26, 0.45]**	**0.41[0.34, 0.47]**
Thailand (*N* = 20 437)	**3.48[2.96, 4.10]**	**3.09[2.60, 3.67]**	**3.31[2.91, 3.77]**	**0.48[0.42, 0.54]**	**0.35[0.30, 0.40]**	**0.41[0.37, 0.45]**	**2.65[2.19, 3.20]**	**2.61[2.06, 3.29]**	**2.62[2.24, 3.07]**	**0.37[0.31, 0.42]**	**0.31[0.25, 0.36]**	**0.33[0.29, 0.38]**
WPR												
China (*N* = 13 302)	**1.19[1.02, 1.38]**	1.09[0.93, 1.28]	1.15[0.99, 1.32]	**0.13[0.02, 0.24]**	0.06[**−**0.05, 0.17]	0.10[**−**0.01, 0.20]	**1.38[1.22, 1.56]**	**1.32[1.15, 1.51]**	**1.35[1.21, 1.51]**	**0.24[0.14, 0.34]**	**0.19[0.10, 0.28]**	**0.22[0.13, 0.30]**
Malaysia (*N* = 4091)	**2.26[1.73, 2.95]**	**1.78[1.32, 2.39]**	**2.08[1.68, 2.56]**	**0.38[0.26, 0.49]**	**0.19[0.09, 0.30]**	**0.29[0.20, 0.37]**	**2.24[1.69, 2.96]**	**1.71[1.17, 2.51]**	**2.05[1.64, 2.58]**	**0.40[0.26, 0.53]**	**0.19[0.06, 0.32]**	**0.30[0.20, 0.39]**
Philippines (*N* = 9578)	**1.95[1.75, 2.19]**	**2.30[2.03, 2.60]**	**2.10[1.92, 2.30]**	**0.41[0.34, 0.47]**	**0.45[0.39, 0.51]**	**0.43[0.38, 0.48]**	**2.48[2.17, 2.84]**	**2.31[2.01, 2.66]**	**2.40[2.16, 2.67]**	**0.53[0.46, 0.61]**	**0.43[0.36, 0.50]**	**0.48[0.43, 0.53]**
Vietnam (*N* = 9866)	**1.35[1.26, 1.45]**	**1.24[1.15, 1.33]**	**1.29[1.22, 1.37]**	**0.24[0.18, 0.29]**	**0.15[0.10, 0.21]**	**0.20[0.15, 0.24]**	**1.35[1.24, 1.47]**	**1.36[1.25, 1.48]**	**1.35[1.26, 1.44]**	**0.23[0.17, 0.30]**	**0.22[0.16, 0.29]**	**0.22[0.18, 0.27]**
AMR												
Mexico (*N* = 13 530)	0.58[0.39, 0.87]	0.56[0.43, 0.73]	0.57[0.43, 0.75]	−0.09[−0.16, −0.02]	−0.11[−0.15, −0.06]	−0.10[−0.15, −0.05]	0.84[0.55, 1.28]	0.74[0.51, 1.07]	0.78[0.57, 1.07]	−0.03[−0.10, 0.04]	−0.05[−0.12, 0.01]	−0.04[−0.09, 0.01]
Uruguay (*N* = 5576)	**1.37[1.08, 1.74]**	1.22[0.90, 1.66]	**1.30[1.06, 1.60]**	**0.12[0.03, 0.20]**	0.03[−0.05, 0.12]	**0.07[0.01, 0.14]**	**1.57[1.14, 2.16]**	1.25[0.90, 1.72]	**1.41[1.09, 1.82]**	**0.16[0.05, 0.28]**	0.05[−0.03, 0.14]	**0.10[0.03, 0.18]**
EUR												
Poland (*N* = 7640)	**1.53[1.26, 1.86]**	**1.27[1.09, 1.47]**	**1.39[1.23, 1.56]**	**0.18[0.10, 0.26]**	**0.10[0.03, 0.16]**	**0.14[0.09, 0.19]**	**1.88[1.56, 2.26]**	**1.52[1.28, 1.82]**	**1.66[1.45, 1.89]**	**0.31[0.22, 0.39]**	**0.18[0.10, 0.26]**	**0.23[0.17, 0.29]**
Romania (*N* = 4472)	1.06[0.81, 1.39]	0.94[0.70, 1.27]	1.00[0.82, 1.23]	0.01[−0.08, 0.10]	−0.03[−0.12, 0.05]	−0.02[−0.08, 0.05]	0.96[0.66, 1.39]	0.94[0.64, 1.40]	0.94[0.71, 1.23]	−0.01[−0.14, 0.12]	−0.01[−0.11, 0.09]	−0.01[−0.09, 0.07]
Russian Federation (*N* = 11 321)	1.20[0.97, 1.47]	0.91[0.73, 1.14]	1.04[0.88, 1.23]	0.06[−0.01, 0.13]	−0.03[−0.10, 0.03]	0.01[−0.04, 0.06]	**1.29[1.01, 1.65]**	1.10[0.88, 1.37]	1.15[0.98, 1.36]	**0.09[0.00, 0.17]**	0.05[−0.03, 0.12]	0.05[−0.01, 0.11]
Turkey (*N* = 8900)	**1.20[1.07, 1.34]**	**1.06[0.95, 1.19]**	**1.13[1.04, 1.22]**	**0.10[0.04, 0.16]**	**0.03[**−**0.04, 0.09]**	**0.06[0.02, 0.11]**	**1.20[1.05, 1.37]**	**1.40[1.20, 1.64]**	**1.28[1.16, 1.43]**	**0.11[0.03, 0.18]**	**0.19[0.11, 0.28]**	**0.14[0.09, 0.20]**
Ukraine (*N* = 8092)	**2.06[1.61, 2.62]**	1.26[0.93, 1.72]	**1.63[1.33, 1.99]**	**0.17[0.11, 0.24]**	0.02[−0.03, 0.08]	**0.09[0.04, 0.13]**	1.08[0.78, 1.50]	**1.91[1.28, 2.85]**	**1.43[1.11, 1.84]**	0.02[−0.07, 0.10]	**0.10[0.03, 0.17]**	**0.07[0.02, 0.12]**
EMR												
Egypt (*N* = 20 443)	**1.24[1.15, 1.34]**	**1.12[1.05, 1.20]**	**1.18[1.12, 1.23]**	**0.14[0.09, 0.19]**	**0.08[0.03, 0.12]**	**0.11[0.08, 0.14]**	**1.51[1.39, 1.64]**	**1.48[1.36, 1.61]**	**1.49[1.41, 1.58]**	**0.26[0.21, 0.31]**	**0.26[0.21, 0.31]**	**0.26[0.22, 0.30]**

AMR = Region of the Americas; CI = confidence interval; EMR = Eastern Mediterranean Region; EUR = European Region; GATS = Global Adult Tobacco Survey; SEAR = South-East Asia Region; SHS = secondhand smoke; WPR = Western Pacific Region. Bold values indicate significance level *P* < .05.

^a^RII (Relative Index of Inequality) values estimated from country-specific individual-level generalized linear models adjusted for age group and gender. A value > 1 indicates that: the poor are more likely to be exposed to SHS at home compared with the rich (in case of wealth inequality) and the less educated are more likely to be exposed to SHS at home compared with the more educated (in case of education inequality).

^b^SII (Slope Index of Inequality) values estimated from country-specific individual-level generalized linear models adjusted for age group and gender. A value > 0 indicates that: the poor are more likely to be exposed to SHS at home compared with the rich (in case of wealth inequality) and the less educated are more likely to be exposed to SHS at home compared with the more educated (in case of education inequality).

In 12 of the 15 countries studied (India, Bangladesh, Thailand, China, Malaysia, the Philippines, Vietnam, Uruguay, Poland, Turkey, Ukraine, and Egypt), RII estimates and their CIs were more than 1 and SII estimates and their CIs were more than 0 indicating that in these countries, those with less education are more likely to be exposed to SHS at home compared with the more educated. There was substantial variation between the countries in SHS exposure at home by levels of education. The RII estimates ranged from 1.28 (95% CI 1.16–1.43) in Turkey to 2.65 (95% CI 2.43–2.88) in India, while the SII estimates ranged from 0.07 (95% CI 0.02–0.12) in Ukraine to 0.48 (95% CI 0.43–0.53) in the Philippines.

Table 3 also presents findings of disaggregated analysis by gender for socioeconomic inequalities in SHS exposure at home. The results were in line with the overall observations made above; and for a majority of the countries, no significant gender differences were observed. Significant wealth inequality in SHS exposure at home was observed only among males in China, Uruguay, and Ukraine while significant education inequality in SHS exposure at home was observed only among males in Uruguay and the Russian Federation; and only among females in Ukraine.

### Socioeconomic Inequalities in SHS Exposure at Workplace

Table 4 presents the RII and SII estimates and their 95% CIs, respectively for wealth and education inequality in SHS exposure at workplace. These comparisons are also shown graphically in Supplementary Figure 4. In four of the 15 countries studied (India, Bangladesh, Thailand, and the Philippines), RII estimates and their CIs were more than 1 and SII estimates and their CIs were more than 0 indicating that in these countries, the poor are more likely to be exposed to SHS at workplace compared with the rich. Variation was observed between the countries in SHS exposure at workplace by levels of wealth. The RII estimates ranged from 1.18 (95% CI 1.00–1.40) in Bangladesh to 2.30 (95% CI 1.83–2.90) in Thailand, while the SII estimates ranged from 0.12 (95% CI 0.01–0.23) in Bangladesh to 0.22 (95% CI 0.12–0.31) in the Philippines.

**Table 4.** Socioeconomic Inequality in SHS Exposure at Workplace

Region/country	Wealth inequality						Education inequality					
	RII [95% CI]			SII [95% CI]			RII [95% CI]			SII [95% CI]		
	Males	Females	Total^a^	Males	Females	Total^b^	Males	Females	Total^a^	Males	Females	Total^b^
SEAR												
India (*N* = 12 852)	**1.59 [1.29, 1.96]**	**3.86 [2.21, 6.76]**	**1.70 [1.40, 2.08]**	**0.16[0.09, 0.24]**	**0.20[0.10, 0.29]**	**0.17[0.11, 0.24]**	**2.16[1.76, 2.65]**	**4.74[2.50, 8.99]**	**2.30[1.90, 2.80]**	**0.27[0.20, 0.34]**	**0.24[0.13, 0.35]**	**0.26[0.20, 0.32]**
Bangladesh (*N* = 1704)	1.18 [0.99, 1.39]	1.53 [0.65, 3.62]	**1.18 [1.00, 1.40]**	0.12[−0.01, 0.24]	0.13[−0.18, 0.43]	**0.12[0.01, 0.23]**	**1.37[1.14, 1.63]**	1.32[0.53, 3.28]	**1.36[1.14, 1.63]**	**0.22[0.09, 0.34]**	0.08[−0.20, 0.35]	**0.20[0.08, 0.31]**
Thailand (*N* = 5021)	**2.51 [1.89, 3.35]**	**1.88 [1.30, 2.73]**	**2.30 [1.83, 2.90]**	**0.30[0.20, 0.40]**	**0.14[0.06, 0.23]**	**0.21[0.15, 0.28]**	**3.00[2.21, 4.07]**	**1.94[1.18, 3.19]**	**2.65[2.07, 3.40]**	**0.38[0.27, 0.49]**	**0.13[0.03, 0.22]**	**0.23[0.16, 0.30]**
WPR												
China (*N* = 1859)	0.95 [0.76, 1.18]	1.52 [0.94, 2.47]	1.02 [0.82, 1.26]	−0.05[−0.22, 0.12]	0.22[−0.04, 0.48]	0.03[−0.12, 0.18]	0.97[0.75, 1.25]	1.23[0.71, 2.10]	1.00[0.78, 1.28]	−0.03[−0.22, 0.16]	0.09[−0.16, 0.34]	0.01[−0.16, 0.17]
Malaysia (*N* = 996)	1.02 [0.64, 1.61]	1.23 [0.55, 2.73]	1.07 [0.72, 1.57]	0.01[−0.20, 0.22]	0.07[−0.16, 0.31]	0.04[−0.11, 0.19]	1.47[0.87, 2.51]	1.62[0.60, 4.38]	1.51[0.95, 2.40]	0.17[−0.07, 0.42]	0.14[−0.13, 0.40]	0.16[−0.02, 0.33]
Philippines (*N* = 2152)	**1.87 [1.37, 2.56]**	**2.08 [1.33, 3.27]**	**1.94 [1.49, 2.53]**	**0.25[0.12, 0.39]**	**0.19[0.07, 0.31]**	**0.22[0.12, 0.31]**	**3.81[2.64, 5.50]**	**2.37[1.52, 3.69]**	**3.22[2.39, 4.34]**	**0.51[0.37, 0.65]**	**0.24[0.11, 0.36]**	**0.35[0.25, 0.45]**
Vietnam (*N* = 2419)	**1.27 [1.07, 1.50]**	0.98 [0.67, 1.42]	**1.21 [1.03, 1.42]**	**0.17[0.04, 0.29]**	−0.02[−0.16, 0.12]	0.09[−0.01, 0.19]	**1.31[1.08, 1.58]**	**1.43[1.01, 2.05]**	**1.34[1.12, 1.59]**	**0.19[0.05, 0.33]**	0.14[−0.03, 0.31]	**0.17[0.06, 0.29]**
AMR												
Mexico (*N* = 2082)	1.36 [0.79, 2.34]	0.40 [0.13, 1.21]	1.00 [0.58, 1.72]	0.07[−0.06, 0.21]	−0.10[−0.21, 0.01]	−0.02[−0.12, 0.07]	**1.66[1.05, 2.60]**	1.06[0.45, 2.50]	1.46[0.99, 2.17]	**0.12[0.01, 0.23]**	0.01[−0.11, 0.12]	0.07[−0.01, 0.14]
Uruguay (*N* = 1796)	1.43 [0.84, 2.42]	1.39 [0.64, 3.02]	1.42 [0.92, 2.21]	0.08[−0.04, 0.21]	0.04[−0.06, 0.15]	0.06[−0.02, 0.14]	1.41[0.74, 2.69]	2.38[0.99, 5.77]	1.69[0.96, 2.96]	0.07[−0.07, 0.21]	0.12[−0.01, 0.25]	**0.10[0.00, 0.20]**
EUR												
Poland (*N* = 3030)	1.18 [0.93, 1.50]	0.79 [0.49, 1.29]	1.06 [0.85, 1.31]	0.07[−0.03, 0.18]	−0.06[−0.16, 0.05]	0.00[−0.07, 0.08]	**2.47[1.80, 3.38]**	**2.12[1.28, 3.51]**	**2.36[1.82, 3.07]**	**0.39[0.25, 0.53]**	**0.18[0.06, 0.29]**	**0.27[0.18, 0.35]**
Romania (*N* = 1175)	0.93 [0.56, 1.53]	1.20 [0.55, 2.63]	1.02 [0.66, 1.57]	−0.03[−0.22, 0.16]	0.06[−0.18, 0.29]	0.01[−0.14, 0.16]	1.75[0.93, 3.28]	1.19[0.60, 2.35]	1.45[0.91, 2.31]	0.19[0.00, 0.39]	0.05[−0.15, 0.25]	0.12[−0.03, 0.26]
Russian Federation (*N* = 5464)	1.04 [0.83, 1.32]	0.97 [0.68, 1.37]	1.01 [0.82, 1.25]	0.02[−0.08, 0.13]	−0.01[−0.10, 0.08]	0.00[−0.07, 0.07]	**1.97[1.51, 2.57]**	1.32[0.84, 2.06]	**1.73[1.36, 2.19]**	**0.32[0.20, 0.44]**	0.07[−0.05, 0.18]	**0.17[0.08, 0.25]**
Turkey (*N* = 2160)	0.81 [0.61, 1.07]	0.64 [0.33, 1.25]	0.78[0.61, 1.00]	−0.08[−0.18, 0.02]	−0.13[−0.31, 0.05]	−0.09[−0.18, 0.00]	**1.79[1.37, 2.34]**	1.57[0.87, 2.86]	**1.74[1.36, 2.23]**	**0.24[0.13, 0.36]**	0.14[−0.04, 0.32]	**0.21[0.12, 0.31]**
Ukraine (*N* = 2761)	**1.35 [1.02, 1.78]**	1.20 [0.73, 1.98]	**1.32[1.02, 1.70]**	**0.13[0.01, 0.25]**	0.04[−0.07, 0.16]	0.08[−0.01, 0.17]	**2.38[1.65, 3.42]**	1.21[0.66, 2.24]	**1.93[1.41, 2.64]**	**0.34[0.21, 0.48]**	0.04[−0.09, 0.17]	**0.16[0.06, 0.25]**
EMR												
Egypt (*N* = 4490)	1.05 [0.92, 1.19]	1.07 [0.82, 1.39]	1.05[0.94, 1.17]	0.03[−0.05, 0.11]	0.04[−0.12, 0.20]	0.03[−0.04, 0.10]	1.14[0.98, 1.32]	1.36[0.99, 1.86]	**1.17[1.02, 1.33]**	0.08[−0.01, 0.18]	0.17[−0.01, 0.34]	**0.10[0.02, 0.18]**

AMR = Region of the Americas; CI = confidence interval; EMR = Eastern Mediterranean Region; EUR = European Region; GATS = Global Adult Tobacco Survey; SEAR = South-East Asia Region; SHS = secondhand smoke; WPR = Western Pacific Region. Bold values indicate significance level *P* < .05.

^a^RII (Relative Index of Inequality) values estimated from country-specific individual-level generalized linear models adjusted for age group and gender. A value > 1 indicates that: the poor are more likely to be exposed to SHS at workplace compared with the rich (in case of wealth inequality) and the less educated are more likely to be exposed to SHS at workplace compared with the more educated (in case of education inequality).

^b^SII (Slope Index of Inequality) values estimated from country-specific individual-level generalized linear models adjusted for age group and gender. A value > 0 indicates that: the poor are more likely to be exposed to SHS at workplace compared with the rich (in case of wealth inequality) and the less educated are more likely to be exposed to SHS at workplace compared with the more educated (in case of education inequality).

In 10 of the 15 countries studied (India, Bangladesh, Thailand, the Philippines, Vietnam, Poland, the Russian Federation, Turkey, Ukraine, and Egypt), RII estimates and their CIs were more than 1 and SII estimates and their CIs were more than 0 indicating that in these countries, those with less education are more likely to be exposed to SHS at workplace compared with the more educated. Substantial variation was observed between the countries in SHS exposure at workplace by levels of education. The RII estimates ranged from 1.17 (95% CI 1.02–1.33) in Egypt to 3.22 (95% CI 2.39–4.34) in the Philippines, while the SII estimates ranged from 0.10 (95% CI 0.02–0.18) in Egypt to 0.35 (95% CI 0.25–0.45) in the Philippines.

Table 4 also presents findings of disaggregated analysis by gender for socioeconomic inequalities in SHS exposure at workplace. In Bangladesh, wealth inequality in SHS exposure at workplace was not observed among males and females independently, while education inequality was observed only among males in Bangladesh, the Russian Federation, Turkey, and Ukraine but not among females; and was not observed among either males or females in Egypt. However, in almost half of the countries, the results were in conformity with the overall observations made above with no significant gender differences.

## Discussion

Our study of socioeconomic inequalities in SHS exposure at homes and at workplaces in LMIC settings indicates that the poor are more likely to be exposed to SHS at home than the rich in 11 out of the 15 countries studied. The association was not so consistent between being poor and exposure to SHS at workplace, and was observed only in four out of the 15 countries studied. Less educated participants were consistently more likely to be exposed to SHS at home and at the workplace. Despite the observed gender differences in SHS exposure (particularly at the workplace) in the LMIC settings studied, and as reported in earlier studies,^4,6^ we did not find significant gender differences in socioeconomic inequalities in SHS exposure at home in the majority of countries studied. In case of SHS exposure at the workplace, we found some evidence that education inequality was observed only among males in some of the countries studied; however, in almost half of the countries studied, there were no significant gender differences.

Our key findings are consistent with limited available data from LMIC settings. A study assessing correlates of SHS exposure at home among nonsmoking adults in Bangladesh suggested that groups with lower educational attainment and literacy were more than twice as likely to be exposed to SHS at home than groups with higher educational attainment.^19^ A study conducted in Vietnam using GATS data suggested that participants who had attained tertiary, high school, and secondary education were 60%, 40%, and 30% less likely to be exposed to SHS at home, respectively, as compared with those who had attained only primary education.^20^ Another study conducted with adult participants in rural China showed that participants who did not complete high school education and who had low income were more likely to be exposed to SHS at home.^21^ Similar findings have been reported in studies from HICs.^3,22,23^

Palipudi et al.^6^ studied the socioeconomic determinants of active tobacco use in 13 GATS countries (excluding Malaysia and Romania which have been included in our analyses) and concluded that current tobacco use (including current smoking or smokeless tobacco use, either daily or occasionally) increased with decrease in education levels in India, Bangladesh, Thailand, the Philippines, and Egypt; however there was an inverse association between tobacco use and education levels in Turkey. We found that SHS exposure at home increased with decrease in education levels in 12 countries including the five countries mentioned above for current tobacco use, as well as Turkey. The more consistent social patterning of SHS exposure (than active tobacco use) across countries found here may be explained by differences in social norms around exposing others to SHS in different socioeconomic groups within these settings.

### Strengths and Limitations

Our study is based on findings from large nationally representative datasets from 15 LMICs where the majority of the world’s smokers reside. We present RII and SII estimates as these are considered to be more robust measures of socioeconomic inequality compared with *AOR*s.^15,16^ Our study focused on socioeconomic inequalities in exposure to SHS at home and indoor workplaces as these are the two settings in which SHS exposure predominantly occurs.^3^ For the latter, we restricted our sample by excluding participants working exclusively outdoors (eg, farmers and outdoor laborers), students, homemakers, the retired, and the unemployed. This may partly explain the absence of a socioeconomic gradient in SHS exposure in the workplace in several LMICs studied. We were unable to examine occupation-based measures of SES as GATS provides limited information about occupational grades. Further, in the case of education variable, we merged “no formal education” with the next higher category “up to primary level” because participants in that category accounted for less than 10% of the study sample for a majority of the countries studied. For remaining countries, in which the percentage of participants in the “no formal education” category was more than 10%, we conducted sensitivity analyses by separating “no formal education” and “up to primary level” categories and found that our results remained unchanged. The authors acknowledge the heterogeneity that exists among the various LMICs studied in terms of the stage of tobacco epidemic, tobacco control policies, and diverse patterns in socioeconomic inequality; however, the fact that GATS provides uniform data from these countries allows broad comparisons to be drawn across these countries through studies such as ours and those previously published.^6^ Data from the first round of GATS is now at least 4 years old and may not reflect the current state of inequalities in SHS exposure, given the fact that tobacco control efforts (particularly smoke-free policies) have been strengthened in some settings. We relied on self-reported measures of exposure to SHS at homes and workplaces in the absence of biological markers such as cotinine levels. Although earlier studies indicate good correlation between cotinine levels and self-reported measures,^24^ more recent studies suggest that self-reported measures of SHS exposure at home and at workplace often underestimate the true prevalence of SHS exposure in the absence of biomarkers such as serum cotinine.^25^ Future studies in LMICs should examine changes in SHS exposure and related socioeconomic inequalities over time and/or assess pre–post smoke-free legislation implementation changes.

### Policy Implications

Our results show that SHS exposure at homes and at workplaces is high in a majority of the LMIC settings studied, reflecting considerable variation between countries. The study indicates that socioeconomic inequalities exist in exposure to SHS at homes as well as at workplaces (to some extent) in these settings. Nearly 71% of middle-income and 88% of low-income countries are still not protected by comprehensive smoke-free policies.^26^ Earlier studies have shown that voluntary, regional, or partial smoke-free policies are not likely to be effective and will often be equity negative.^27,28^ Reasonably good evidence suggests that comprehensive smoke-free policies have an equity positive or neutral effect on health outcomes strongly influenced by SHS exposure. For example, recent work has shown that comprehensive smoke-free legislation in England was associated with a greater reduction in admissions for respiratory tract infections in children from lower SES groups.^29^ However, comprehensively enforced smoke-free policies may be less likely to be implemented in low SES settings.^22^ To address socioeconomic inequalities in SHS exposure at work, there is a need for accelerated implementation of comprehensive smoke-free policies.^28^ Addressing inequalities in SHS exposure at home would require addressing both inequalities in the prevalence of smoking and inequalities in social norms about exposing nonsmokers to SHS. To reduce inequalities in smoking, the implementation of tobacco control policies needs to be strengthened particularly interventions that have been shown to be pro-equity, such as increasing tobacco taxation.^30^ Focused efforts are required to address social norms around exposing others to SHS (eg, awareness through mass media campaigns and other educational interventions), targeting the socioeconomically disadvantaged groups. Smoke-free policies have been shown to have a positive influence on social norms concerning SHS exposure at home.^8^

## Conclusion

SHS exposure at home is higher among the socioeconomically disadvantaged groups (the poor and the less educated) in a majority of the LMICs studied. SHS exposure at workplace is higher among the less educated groups in a majority of the LMICs studied. Accelerated implementation of pro-equity tobacco control interventions, including increased taxation, along with targeted efforts among the socioeconomically disadvantaged groups are needed to reduce inequalities in SHS exposure in LMICs.

## Supplementary Material

Supplementary Figures 1–4 and Tables 1 and 2 can be found online at http://www.ntr.oxfordjournals.org

## Funding

This work was supported by a Wellcome Trust Capacity Strengthening Strategic Award to the Public Health Foundation of India and a consortium of UK universities. CM is funded by a NIHR Research Professorship award. The funding bodies had no involvement in the study design; collection, analysis, and interpretation of data; and decision to submit the article for publication.

## Declaration of Interests

*None declared.*

## Supplementary Material

Supplementary Data
