# Supplementary material for: Socioeconomic Inequalities in Secondhand Smoke Exposure at Home and at Work in 15 Low- and Middle-Income Countries
Source: Nicotine Tob Res. 2015 Nov 25;18(5):1230–9. doi: 10.1093/ntr/ntv261 (PMC4826490; doi:10.1093/ntr/ntv261)
Supplement: Supplementary Data [file supp_18_5_1230__index.html]

Socioeconomic Inequalities in Secondhand Smoke Exposure at Home and at Work in Fifteen Low- and Middle-Income Countries — Socioeconomic Inequalities in Secondhand Smoke Exposure at Home and at Work in 15 Low- and Middle-Income Countries — Socioeconomic Inequalities in Secondhand Smoke Exposure at Home and at Work in 15 Low- and Middle-Income Countries — Supplementary Data 

# Socioeconomic Inequalities in Secondhand Smoke Exposure at Home and at Work in 15 Low- and Middle-Income Countries

## Supplementary Data

Data files

- Supplementary Data - Supplementary Data
- Supplementary Data - Supplementary Data
- Supplementary Data - Supplementary Data
- Supplementary Data - Supplementary Data
- Supplementary Data - Supplementary Data
- Supplementary Data - Supplementary Data
- Supplementary Data - Supplementary Data
