# Supplementary figures and images for: Socioeconomic Inequalities in Secondhand Smoke Exposure at Home and at Work in 15 Low- and Middle-Income Countries
Source: Nicotine Tob Res. 2015 Nov 25;18(5):1230–9. doi: 10.1093/ntr/ntv261 (PMC4826490; doi:10.1093/ntr/ntv261)

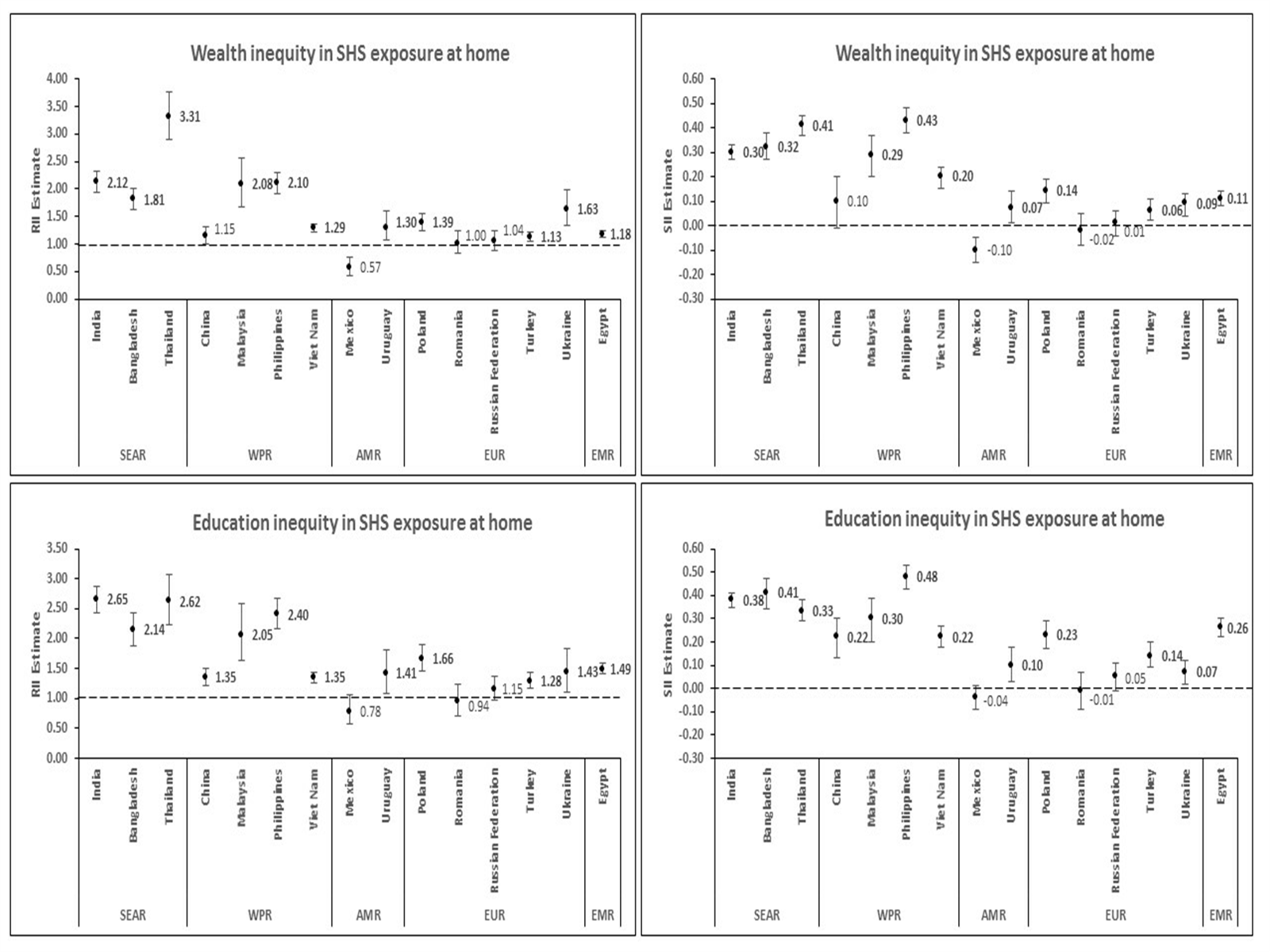

Supplement: Supplementary Data [file supp_ntv261_Supplementary_figure_3_1200dpi.tif]

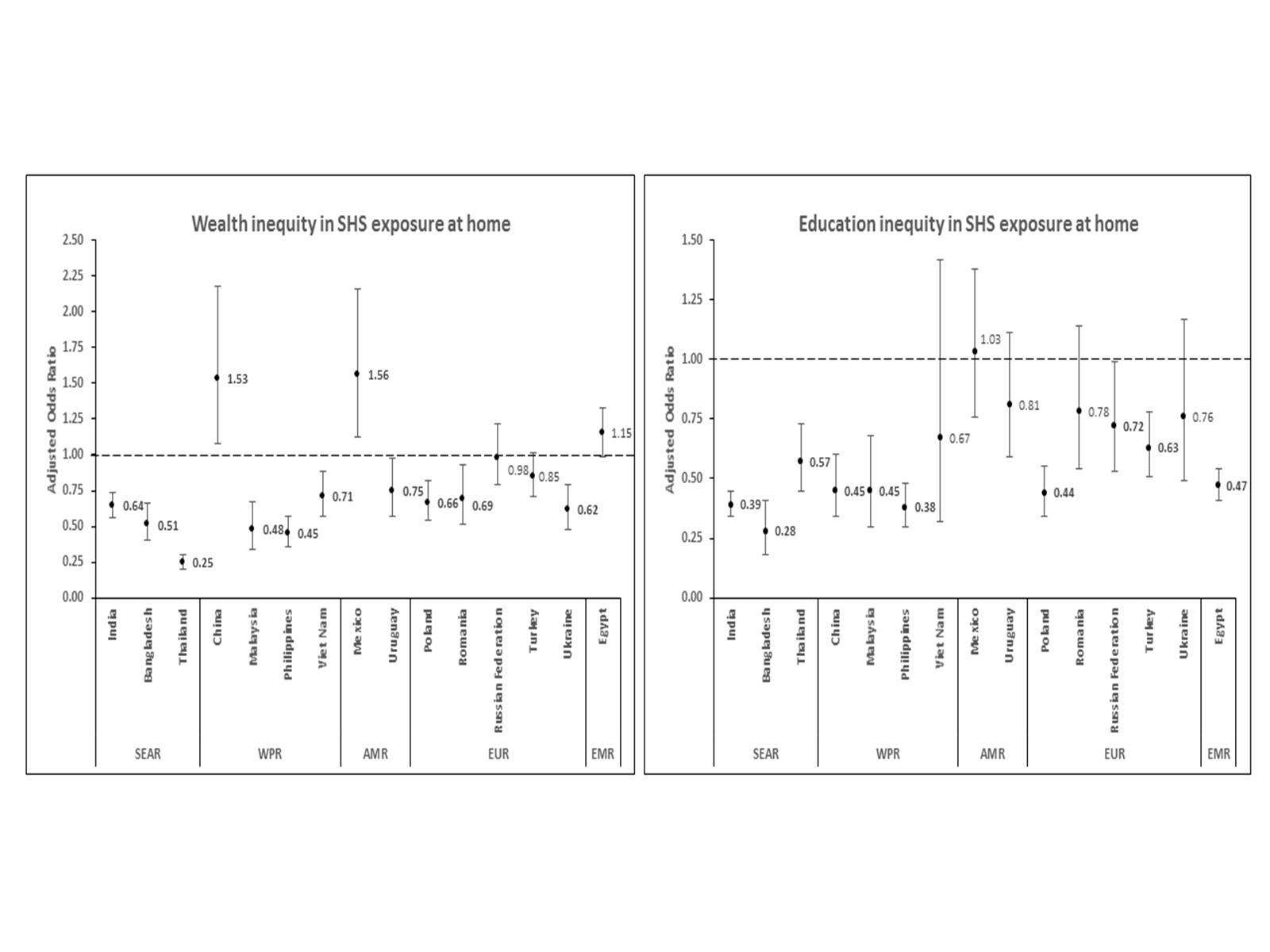

Supplement: Supplementary Data [file supp_ntv261_Supplementary_figure_1_1200dpi_01_.tif]

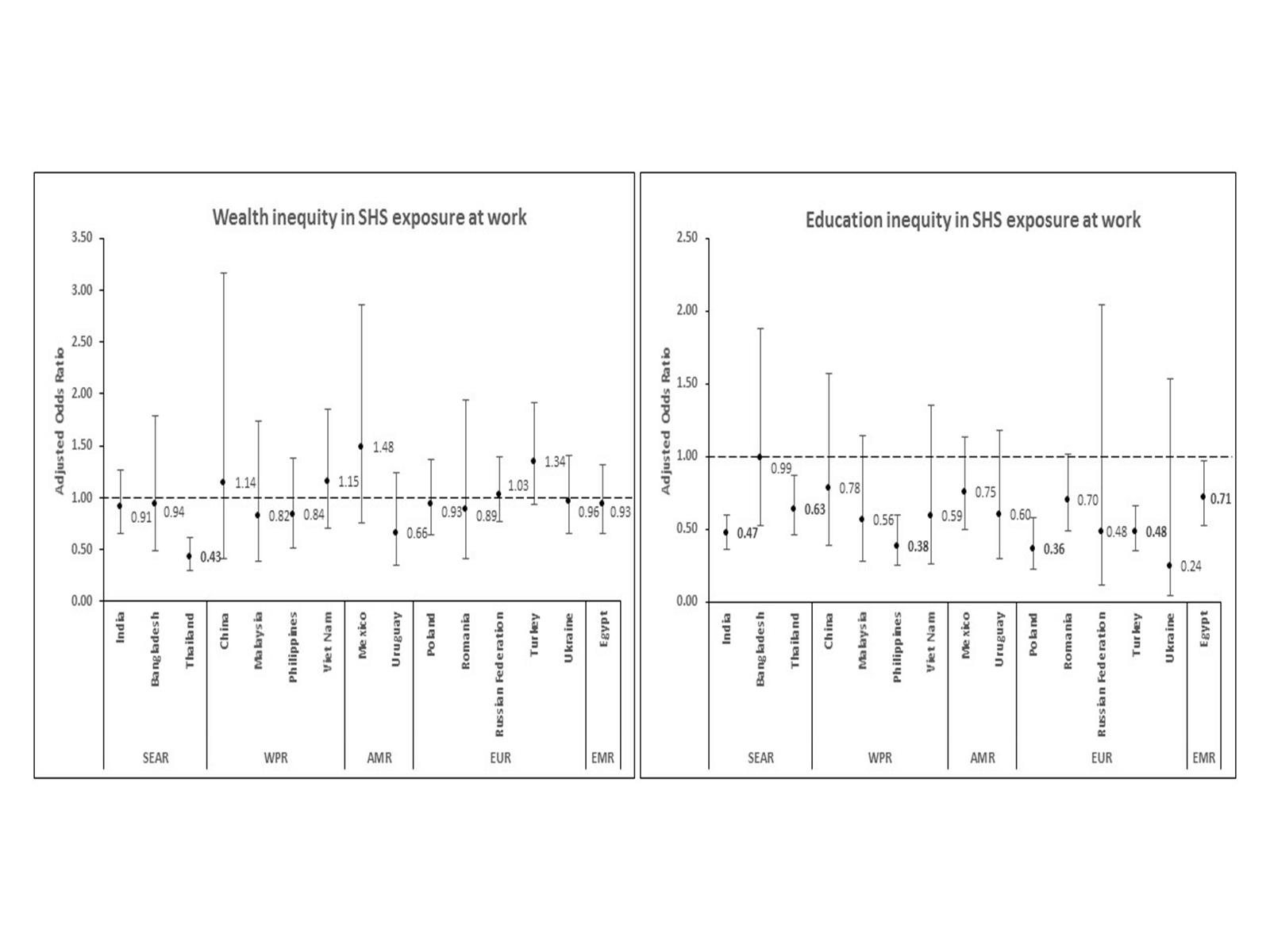

Supplement: Supplementary Data [file supp_ntv261_Supplementary_Figure_2_1200dpi_01_.tif]

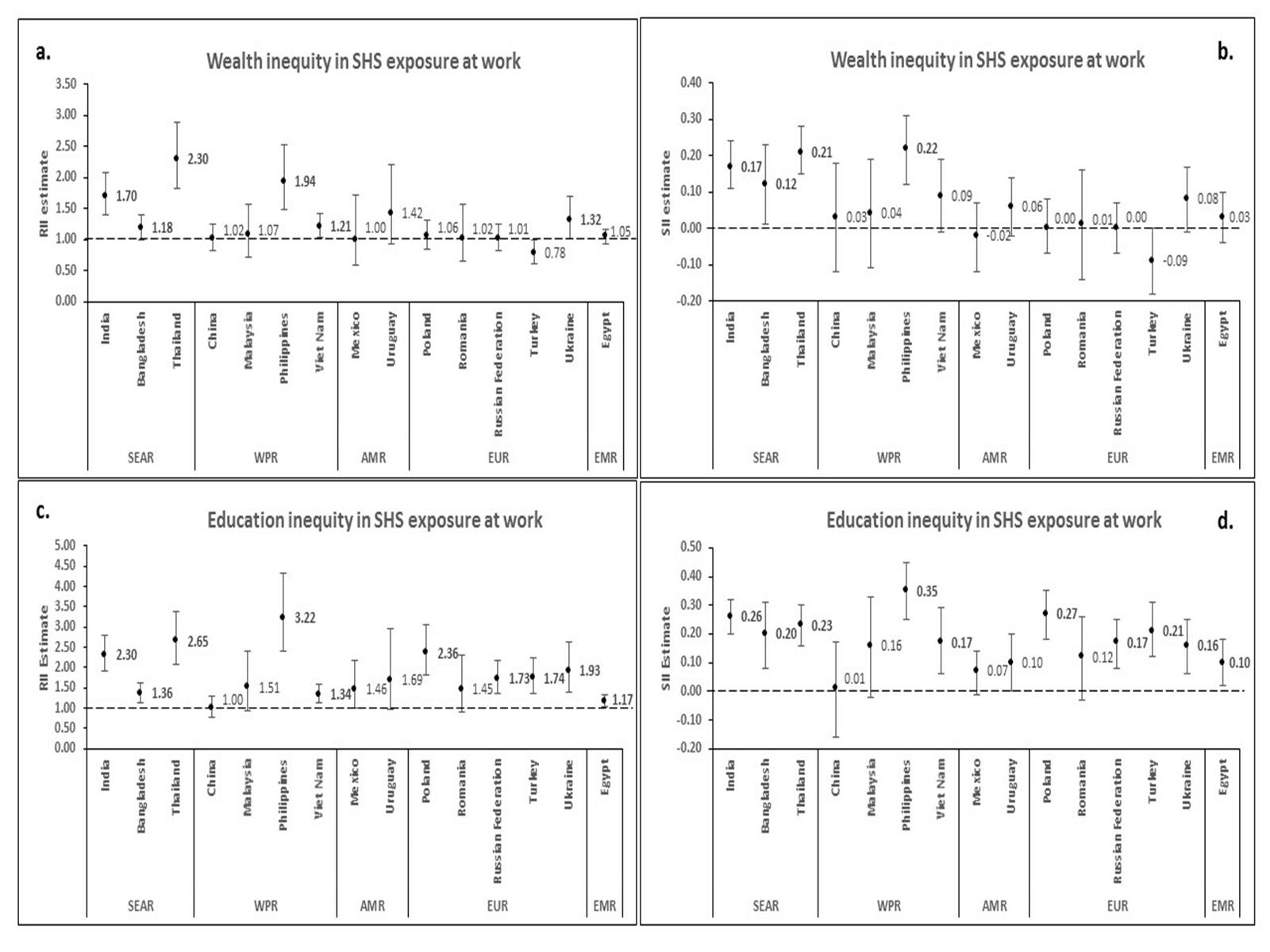

Supplement: Supplementary Data [file supp_ntv261_Supplementary_figure_4_1200dpi_01_.tif]
