# Supplementary material for: Socioeconomic Inequalities in Secondhand Smoke Exposure at Home and at Work in 15 Low- and Middle-Income Countries
Source: Nicotine Tob Res. 2015 Nov 25;18(5):1230–9. doi: 10.1093/ntr/ntv261 (PMC4826490; doi:10.1093/ntr/ntv261)
Supplement: Supplementary Data [file supp_ntv261_Supplementary_Table_2_Missing_Data.docx]

| **Supplementary Table 2: Missing data for SHS exposure at Workplace among GATS participants (2008-2011)**  ***N (%)*** | | | | | | | | | | | | | | | |
| --- | --- | --- | --- | --- | --- | --- | --- | --- | --- | --- | --- | --- | --- | --- | --- |
|  | **SEAR** | | | **WPR** | | | | **AMR** | | **EUR** | | | | | **EMR** |
|  | **India**  **N=12,852** | **Bangladesh**  **N=1,704** | **Thailand**  **N=5,021** | **China**  **N=1,859** | **Malaysia**  **N=996** | **Philippines**  **N=2,152** | **Viet Nam**  **N=2,419** | **Mexico**  **N=2,082** | **Uruguay**  **N=1,796** | **Poland**  **N=3,030** | **Romania**  **N=1,175** | **Russian Federation**  **N=5,464** | **Turkey**  **N=2,160** | **Ukraine**  **N=2,761** | **Egypt**  **N=4,490** |
| ***Dependent variable*** | | | | | | | | | | | | | | | |
| SHS exposure at workplace* | 667  (4.9) | 104  (5.7) | 51  (1.0) | 10  (0.5) | 122  (10.9) | 17  (0.8) | 21  (0.9) | 17  (0.8) | 2  (0.1) | 70  (2.3) | 16  (1.3) | 114  (2.0) | 11  (0.5) | 52  (1.8) | 26  (0.6) |
| ***Independent variables*** | | | | | | | | | | | | | | | |
| Age group | - | - | - | - | - | - | - | - | - | - | - | - | - | - | - |
| Gender | - | - | - | - | - | - | - | - | - | - | - | - | - | - | - |
| Residence | - | - | - | - | - | - | - | - | - | - | - | - | - | - | - |
| Education | 3  (0.02) | 4  (0.2) | 10  (0.2) | 2  (0.1) | 6  (0.5) | - | 1  (0.04) | 2  (0.1) | - | 4  (0.1) | 1  (0.1) | 1  (0.01) | - | 1  (0.03) | - |
| Wealth Quintile | - | - | - | - | - | - | - | - | 1  (0.05) | - | - | - | - | - | - |
| Occupation | - | - | - | - | - | - | - | - | - | - | - | - | - | - | - |
| ***Total missing cases*** | ***670***  ***(4.9)*** | ***108***  ***(6.0)*** | ***61***  ***(1.2)*** | ***12***  ***(0.6)*** | ***128***  ***(11.4)*** | ***17***  ***(0.8)*** | ***22***  ***(0.9)*** | ***19***  ***(0.9)*** | ***3***  ***(0.2)*** | ***74***  ***(2.4)*** | ***17***  ***(1.4)*** | ***115***  ***(2.1)*** | ***11***  ***(0.5)*** | ***53***  ***(1.9)*** | ***26***  ***(0.6)*** |

*Participants who responded “Don’t know” or “Refused to answer” for the question “During the past 30 days, did anyone smoke in the indoor areas where you work?” were dropped from the analysis.
