# Supplementary material for: Socioeconomic Inequalities in Secondhand Smoke Exposure at Home and at Work in 15 Low- and Middle-Income Countries
Source: Nicotine Tob Res. 2015 Nov 25;18(5):1230–9. doi: 10.1093/ntr/ntv261 (PMC4826490; doi:10.1093/ntr/ntv261)
Supplement: Supplementary Data [file supp_ntv261_SUPPLEMENTARY_FIGURE_TITLES.docx]

**SUPPLEMENTARY FIGURE TITLES**

**Supplementary Figure 1:** Adjusted Odds Ratio estimates showing SHS exposure at home [highest wealth quintile vs. lowest (ref.) and highest eduction level vs. lowest (ref.)]

**Supplementary Figure 2:** Adjusted Odds Ratio estimates showing SHS exposure at workplace [highest wealth quintile vs. lowest (ref.) and highest eduction level vs. lowest (ref.)]

**Supplementary Figure 3:** Socioeconomic inequality in SHS exposure at home --- a) RII estimates of wealth inequality in SHS exposure at home; b) SII estimates of wealth inequality in SHS exposure at home; c) RII estimates of education inequality in SHS exposure at home; and d) SII estimates of education inequality in SHS exposure at home

**Supplementary Figure 4:** Socioeconomic inequality in SHS exposure at workplace --- a) RII estimates of wealth inequality in SHS exposure at work; b) SII estimates of wealth inequality in SHS exposure at work; c) RII estimates of education inequality in SHS exposure at work; and d) SII estimates of education inequality in SHS exposure at work
