# Supplementary material for: Socioeconomic Inequalities in Secondhand Smoke Exposure at Home and at Work in 15 Low- and Middle-Income Countries
Source: Nicotine Tob Res. 2015 Nov 25;18(5):1230–9. doi: 10.1093/ntr/ntv261 (PMC4826490; doi:10.1093/ntr/ntv261)
Supplement: Supplementary Data [file supp_ntv261_Supplementary_Table_1_Missing_Data.docx]

| **Supplementary Table 1: Missing data for SHS exposure at Home among GATS participants (2008-2011)**  ***N (%)*** | | | | | | | | | | | | | | | |
| --- | --- | --- | --- | --- | --- | --- | --- | --- | --- | --- | --- | --- | --- | --- | --- |
|  | **SEAR** | | | **WPR** | | | | **AMR** | | **EUR** | | | | | **EMR** |
|  | **India**  **N=67,006** | **Bangladesh**  **N=9,323** | **Thailand**  **N=20,437** | **China**  **N=13,302** | **Malaysia**  **N=4,091** | **Philippines**  **N=9,578** | **Viet Nam**  **N=9,866** | **Mexico**  **N=13,530** | **Uruguay**  **N=5,576** | **Poland**  **N=7,640** | **Romania**  **N=4,472** | **Russian Federation**  **N=11,321** | **Turkey**  **N=8,900** | **Ukraine**  **N=8,092** | **Egypt**  **N=20,443** |
| ***Dependent variable*** | | | | | | | | | | | | | | | |
| SHS exposure at home* | 2041 (2.9) | 229  (2.4) | 84  (0.4) | 33  (0.2) | 140  (3.3) | 121  (1.2) | 55  (0.5) | 27  (0.2) | 2  (0.03) | 151  (1.9) | 16  (0.4) | 69  (0.6) | 118  (1.3) | 37  (0.4) | 470  (2.2) |
| ***Independent variables*** | | | | | | | | | | | | | | | |
| Age group | - | - | - | - | - | - | - | - | - | - | - | - | - | - | - |
| Gender | - | - | - | - | - | - | - | - | - | - | - | - | - | - | - |
| Residence | - | - | - | - | - | - | - | - | - | - | - | - | - | - | - |
| Education | 137  (0.2) | 64  (0.7) | 40  (0.2) | 5  (0.04) | 17  (0.4) | 1  (0.01) | 4  (0.04) | 26  (0.2) | - | 22  (0.3) | 25  (0.5) | 3  (0.03) | 12  (0.1) | 16  (0.2) | 6  (0.03) |
| Wealth Quintile | 1  (0.001) | 1  (0.01) | - | - | - | - | - | 18  (0.1) | 3  (0.05) | - | - | - | - | - | 1  (0.0) |
| Occupation | 111  (0.2) | 12  (0.1) | 5  (0.02) | 14  (0.1) | 2  (0.05) | 1  (0.01) | - | 16  (0.1) | - | 27  (0.3) | 4  (0.1) | 13  (0.1) | 0 | 13  (0.2) | (0.02) |
| ***Total missing cases*** | ***2290 (3.3)*** | ***306***  ***(3.2)*** | ***129***  ***(0.6)*** | ***52***  ***(0.4)*** | ***159***  ***(3.7)*** | ***123***  ***(1.3)*** | ***59***  ***(0.6)*** | ***87***  ***(0.6)*** | ***5***  ***(0.1)*** | ***200***  ***(2.5)*** | ***45***  ***(1.0)*** | ***85***  ***(0.7)*** | ***130***  ***(1.4)*** | ***66***  ***(0.8)*** | ***481***  ***(2.3)*** |

*Participants who responded “Don’t know” or “Refused to answer” for the question “How often does anyone smoke inside your home?” were dropped from the analysis.
